# *In vitro* and *in vivo* antioxidant activities of inulin

**DOI:** 10.1371/journal.pone.0192273

**Published:** 2018-02-02

**Authors:** Hong-Mei Shang, Hai-Zhu Zhou, Jun-Yan Yang, Ran Li, Hui Song, Hong-Xin Wu

**Affiliations:** 1 College of Animal Science and Technology, Jilin Agricultural University, Jilin, Changchun, China; 2 Key Laboratory of Animal Nutrition and Feed Science of Jilin Province, Jilin Agricultural University, Jilin, Changchun, China; 3 School of Life Sciences, Jilin Agricultural University, Jilin, Changchun, China; 4 Grassland Research Institute of CAAS, Neimenggu, Hohhot, China; Institute of medical research and medicinal plant studies, CAMEROON

## Abstract

This study was designed to investigate the *in vitro* and *in vivo* antioxidant activities of inulin. The *in vitro* assays demonstrated that the antioxidant activities of inulin, including the DPPH radical scavenging activity, ABTS scavenging activity and ferric reducing power, were weak and significantly lower than those of Vitamin C (*P* < 0.05). The influence of dietary supplementation with inulin on the antioxidant status of laying hens was evaluated with *in vivo* antioxidant assays. The results indicated that inulin supplementation quadratically improved the egg production rate of the laying hens (*P* < 0.01). The antioxidant enzyme activities in the serum, including SOD, CAT, and GSH-Px, and the total antioxidant capacity increased quadratically as inulin levels increased (*P* < 0.001). The levels of MDA in the serum decreased quadratically as inulin levels increased (*P* < 0.001). These findings suggest that inulin has the potential to improve the antioxidant status of laying hens.

## Introduction

Free radicals are part of normal metabolites for many organisms, and a complex system of endogenous and exogenous antioxidant sources in the body are employed to mitigate the potential damage from free radicals [1]. When the body is in a state of aging or stress, these highly reactive chemical species are produced excessively, and structural abnormalities and dysfunction of the cell and mitochondrial membranes can arise [2]. Excessive free radicals affect animal performance, even resulting in the development of diseases [3]. Improving the antioxidant status of living animals is one of the primary methods for improving bird performance in the poultry production industry [4, 5].

Antioxidants are a class of chemical substances that reduce free radicals and inhibit oxidation directly or indirectly. Oxidative stress in the body may be alleviated by exogenous supplementation with antioxidants. However, several synthetic antioxidants have shown potential adverse effects, such as liver injury and carcinogenesis, especially long-term administration of synthetic antioxidants [6]. Thus, exploration of safe and natural antioxidants to resist oxidative stress has become a research hotspot in recent years.

Inulin is a type of fructosan, extracted primarily from plant organs, such as the tubers of dahlia and the roots of elecampane, chicory and Jerusalem artichoke [7, 8]. Inulin is a linear polymer polysaccharide in which D-fructose is joined by the linkage of a -(2→1) glycosidic bond in the molecular structure, and the typical terminal is a D-glucose molecule [9, 10]. Because of the glycosidic bond types, inulin cannot be digested by digestive enzymes of monogastric animals. However, inulin can be utilized by the beneficial bacteria in the large intestine [11]. Currently, because of the biocompatible and biodegradable properties of the human body, inulin and its derivatives have been widely used in food and pharmaceutical industries [12, 13]. Generally, inulin is used as a texture modifier, fat replacer, sugar substitute, and prebiotic in food and pharmaceutical industries [14]. However, few studies have investigated the antioxidant activity of inulin. To further clarify the activities of inulin, the present study was conducted to research the *in vitro* and *in vivo* antioxidant activities of inulin. The *in vivo* antioxidant study was designed to assess the effects of supplementation with inulin on the antioxidant status of laying hens.

## Materials and methods

### Materials and reagents

Inulin from Jerusalem artichoke root was purchased from Beijing Wede biological Technology Co., Ltd. (Beijing, China). The average polymerization degree of inulin was approximately 20 fructosyl fructose units. The reagents including 1,1-diphenyl-2-picrylhydrazyl (DPPH) radical, Vitamin C and 2,2-azino-bis-3-ethyl-benzothiazoline-6-sulfonic acid) (ABTS) radical were obtained from Sigma-Aldrich (St. Louis, USA). All other chemicals used were analytical grade and bought from local suppliers.

### *In vitro* antioxidant activity assay

#### DPPH radical scavenging assay

In this study, the DPPH radical scavenging activity of inulin was determined by the colorimetric method as described previously [15] with several modifications. Briefly, 1.0 mL of 0.1 mM DPPH solution in ethanol was prepared and was mixed with 3.0 mL of 0.25–10 mg/mL of inulin. The mixture was placed at room temperature. After incubation for 30 min, the mixture was determined at the wavelength of 517 nm against ethanol. The DPPH radical scavenging activity of inulin was computed using the formula below:

$$\mathrm{DPPH}\;\mathrm{radical}\;\mathrm{scavenging}\;\mathrm{activity}\;(\%)=\frac{[A_{0}-(A_{1}-A_{2})]\times 100}{A_{0}}$$ (1)

where *A*_0_ was the absorbance of the control group (water instead of the sample solution). *A*_1_ was the absorbance of the test group. *A*_2_ was the absorbance of the sample (ethanol instead of the DPPH solution). Vitamin C was used as the positive control.

#### ABTS radical scavenging assay

The ABTS radical scavenging activity of inulin was evaluated using a previously described procedure [16] with slight modifications. Vitamin C served as the positive reference reagent. The ABTS radical (ABTS^+^) was produced with the mixture of 5 mL of ABTS solution (7 mM) and 1 mL of potassium persulfate aqueous solution (15 mM) for 24 h in darkness at 20°C. After incubation, the mixture was diluted with deionized water to obtain an absorbance of 0.700±0.002 at a wavelength of 734 nm. Next, 3 mL of the diluted solution was reacted with 0.75 mL of various concentrations (0.25–10 mg/mL) of inulin solutions. The mixture was reacted for 15 min at 20°C before the absorbance at 734 nm was determined against distilled water. The control contained all of the reagents but distilled water instead of the inulin solution, and the absorbance was determined as *A*_0_. *A*_1_ was the reaction result for inulin. *A*_2_ was the result of all the reagents with the ABTS solution used instead of distilled water. The ABTS radical scavenging activity of inulin was calculated by the equation below:

$$\mathrm{ABTS}\;\mathrm{radical}\;\mathrm{scavenging}\;\mathrm{activity}\;(\%)=\frac{[A_{0}-(A_{1}-A_{2})]\times 100}{A_{0}}$$ (2)

#### Ferric reducing power

The ferric reducing power of inulin was determined with a reported procedure [17] with minor modifications. Vitamin C served as the positive reference reagent. First, 1.5 mL of the inulin solutions of 0.25 to 4 mg/mL concentrations were mixed with 1.5 mL of sodium phosphate buffer (0.2 M, pH 6.6) and 1.5 mL of potassium ferricyanide (1%, w/v). The mixture was reacted at 50°C for 20 min. After that step, the mixture was cooled quickly. After addition of 1.5 mL of trichloroacetic acid (TCA, 10%, w/v), the mixture was centrifuged at 3000×g for 10 min. After centrifugation, 1.5 mL of the centrifugate was pulled out and was mixed thoroughly with 1.5 mL of deionized water and 0.3 mL of ferric chloride (0.1%, w/v). The absorbance of the mixture was determined at a wavelength of 700 nm against distilled water. The higher the absorbance of the reaction mixture, the stronger the reducing power of the sample. The ferric reducing power of inulin was calculated by the equation below:

$$\mathrm{Ferric}\;\mathrm{reducing}\;\mathrm{power}=A_{1}-A_{2}$$ (3)

where *A*_1_ was the absorbance of the inulin sample. *A*_2_ was the result of all the reagents with the FeCl_3_ solution used instead of the distilled water.

### *In vivo* antioxidant activity

#### Animal design

The experimental procedures used in the feeding study were approved by the Animal Care and Use Committee of Jilin Agricultural University. Three hundred thirty-week-old Hy-Linen brown commercial laying hens were randomly divided into 5 treatment groups, and each group had 6 replicates of 10 birds. All diets were formulated to meet the nutrient requirements for laying hens [18]. Diets had 5, 10, 15, or 20 g/kg of inulin added in crushed form. The feeding experiment lasted 8 weeks. The ingredients of the laying hens’ diet are shown in Table 1. Feed and water were supplied *ad libitum*. The laying hens were raised individually in wire cages (45×45×50 cm) in a temperature controlled room (22 ± 2°C). Light was provided 16 h per day.

**Table 1:** Ingredients and nutrient concentrations of the diet (g/kg as fed unless noted).

Item	Dietary inulin concentration (g/kg)				
	0 (Control)	5	10	15	20
**Ingredient**					
**Corn**	602.0	602.0	602.0	602.0	602.0
**Soybean meal**	240.0	240.0	240.0	240.0	240.0
**Soybean oil**	10.0	10.0	10.0	10.0	10.0
**Fish meal**	22.4	22.4	22.4	22.4	22.4
**Wheat bran**	31.1	26.1	21.1	16.1	11.1
**Inulin**	0	5.0	10.0	15.0	20.0
**Calcium hydrogen phosphate**	10.0	10.0	10.0	10.0	10.0
**Limestone**	70.0	70.0	70.0	70.0	70.0
**Sodium chloride**	3.3	3.3	3.3	3.3	3.3
**DL-Methionine**	1.1	1.1	1.1	1.1	1.1
**L-Lysine**	0.1	0.1	0.1	0.1	0.1
**Premix**	10.0	10.0	10.0	10.0	10.0
**Chemical composition**					
**CP**	186.9	186.7	186.4	186.2	186.1
**Calcium**	33.6	33.7	33.5	33.4	33.6
**Total phosphorus**	6.0	6.0	5.9	5.9	6.0
**Lysine**	10.2	10.2	10.3	10.4	10.2
**Methionine + cysteine**	7.5	7.6	7.5	7.5	7.6
**ME (MJ/kg)**	11.50	11.50	11.51	11.51	11.52

The control group was fed the standard diet. The other treatment diets were supplemented with 5, 10, 15, or 20 g of inulin powder/kg of diet by replacing the equivalent amount of wheat bran in the standard diet formulation.

Premix supplied per kilogram of diet: retinyl palmitate, 3.96 mg; cholecalciferol, 0.07 mg; DL-α-tocopheryl acetate, 20 mg; menadione sodium bisulfite, 4 mg; thiamine mononitrate, 1.63 mg; riboflavin, 8 mg; niacin, 30 mg; pantothenic acid, 10 mg; folic acid, 0.5 mg; biotin, 0.2 mg; choline chloride, 250 mg; cyanocobalamin, 0.012 mg; Cu, 8 mg; Fe, 30 mg; I, 0.6 mg; Mn, 50 mg; Se, 0.12 mg; Zn, 40 mg.

#### Laying performance

The egg production, egg weight and feed consumption were recorded daily for each replicate. Egg mass was count by egg weight times egg production. Feed conversion ratio was calculated by dividing feed consumption by egg mass production.

#### Antioxidant status of laying hens

At the end of the eighth week, 18 laying hens were randomly selected from each treatment (3 hens per replicate), and blood samples were taken from the wing vein into a non-heparinized tube. The blood was allowed to clot at 37°C for 100 min. After that step, the individual blood was separated by centrifugation at 3000×g for 10 min and 4°C to obtain the serum. The serum samples were stored at −20°C until the analysis of antioxidant enzyme activities and concentration of malondialdehyde (MDA). The antioxidant enzymes included superoxide dismutase (SOD), catalase (CAT), and glutathione peroxidase (GSH-Px). The total antioxidant capacity (T-AOC) of serum was also determined. All these indicators were assayed by the colorimetric method and followed the instructions of the kits (Nanjing Jiancheng Bioengineering Institute, Nanjing, China). The antioxidant enzyme activities were expressed as units (U) per milliliter of serum.

### Statistical analysis

All *in vitro* experiments were performed at least in triplicate. Analyses of all samples were run in triplicate and averaged. The resulting values are presented as the mean ± standard deviation (SD). The one-way ANOVA in SPSS 17.0 for Windows (SPSS Inc., Chicago, IL) was used for statistical analysis. Duncan’s post hoc tests were performed when significant differences were found (*P* < 0.05). The influence of inulin doses on indicators was determined using curve estimation for linear and quadratic terms.

## Results and discussion

### Antioxidant activity *in vitro*

#### DPPH radical scavenging activity

DPPH radical scavenging activity is extensively used to assess the antioxidant capacity of biological samples. It is a stable free radical and shows the strongest absorption at a wavelength of 517 nm. In this method, the antioxidant substances are able to reduce the DPPH radical to the yellow diphenyl-picrylhydrazine [19]. The relationship between the antioxidant ability of the sample and the yellow color compound content in the reaction system is linear and dose-dependent. Therefore, the antioxidant activity of a sample can be expressed as its ability to scavenge the DPPH radical.

**Fig 1:**
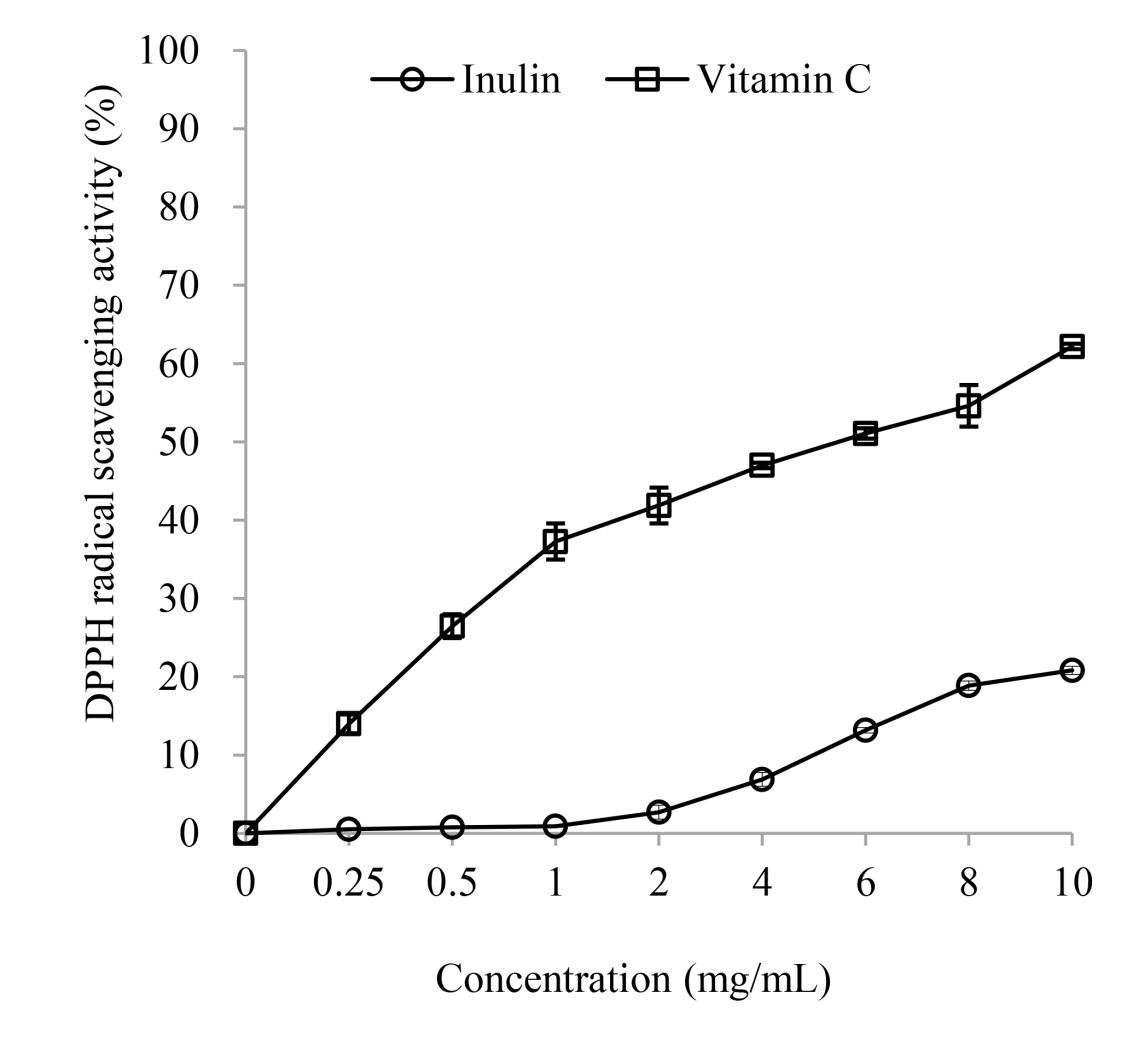
DPPH radical scavenging activity of inulin.

The DPPH radical scavenging ability of inulin is shown in Fig 1. The results indicated that both inulin and Vitamin C increased DPPH radical scavenging activities in a concentration-dependent manner. As the concentration of inulin increased from 0.25 to10 mg/mL, the DPPH radical scavenging activity of inulin increased linearly (*R*^*2*^ = 0.985, *P*.<0.05). The DPPH radical scavenging activity of Vitamin C increased quadratically (*R*^*2*^ = 0.829, *P*<0.05) when the Vitamin C levels increased from 0.25 to10 mg/mL. At a dose of 10 mg/ml, the DPPH radical scavenging activity of inulin was 20.81±0.15%. However, the DPPH scavenging ability of inulin was significantly lower than that of Vitamin C in doses ranging from 0.25 to 10 mg/mL (*P* < 0.05). Therefore, inulin has weaker DPPH radical scavenging activity than Vitamin C *in vitro*.

#### ABTS radical scavenging activity

The ABTS radical scavenging activity method was pioneered by Miller and Rice-Evans (1993) [20]. Through oxidation by oxygen activity, colorless ABTS is transformed to the stable, blue-green ABTS^• +^, which is specifically absorbed at a wavelength of 734 nm. When colored ABTS^• +^ is reacted with any antioxidant ingredient, ABTS^• +^ is reduced to the original colorless ABTS. The ABTS radical cation de-coloration assay only needs a short reaction time (approximately 15 min) and has been extensively used to evaluate the antioxidant activity of biological samples [21].

**Fig 2:**
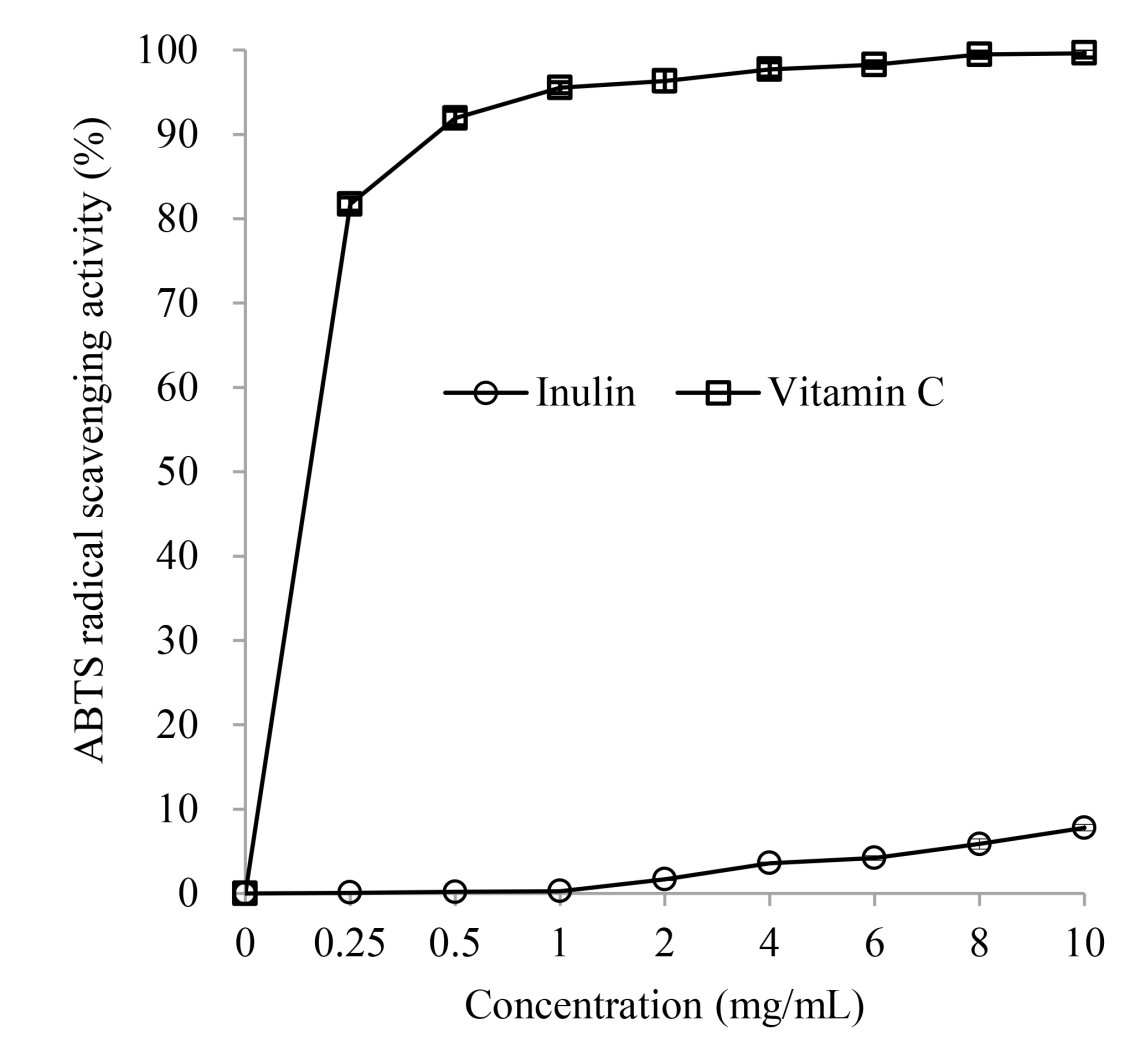
ABTS radical scavenging activity of inulin.

The ABTS radical scavenging ability of inulin is presented in Fig 2. The activity of inulin toward the ABTS radical was related to inulin dosage within the test range (0.25–10 mg/mL). The activity increased linearly (*R*^*2*^ = 0.988, *P*<0.05) as inulin levels increased. At a dose of 10 mg/mL, the scavenging activity of inulin was only 7.79±0.37%. Furthermore, the activity of inulin toward the ABTS radical was significantly lower than that of Vitamin C at each dosage (*P* < 0.05). The results of the ABTS radical cation de-coloration assay indicated that inulin has weaker ABTS radical scavenging activity than Vitamin C *in vitro*.

#### Ferric reducing power

**Fig 3:**
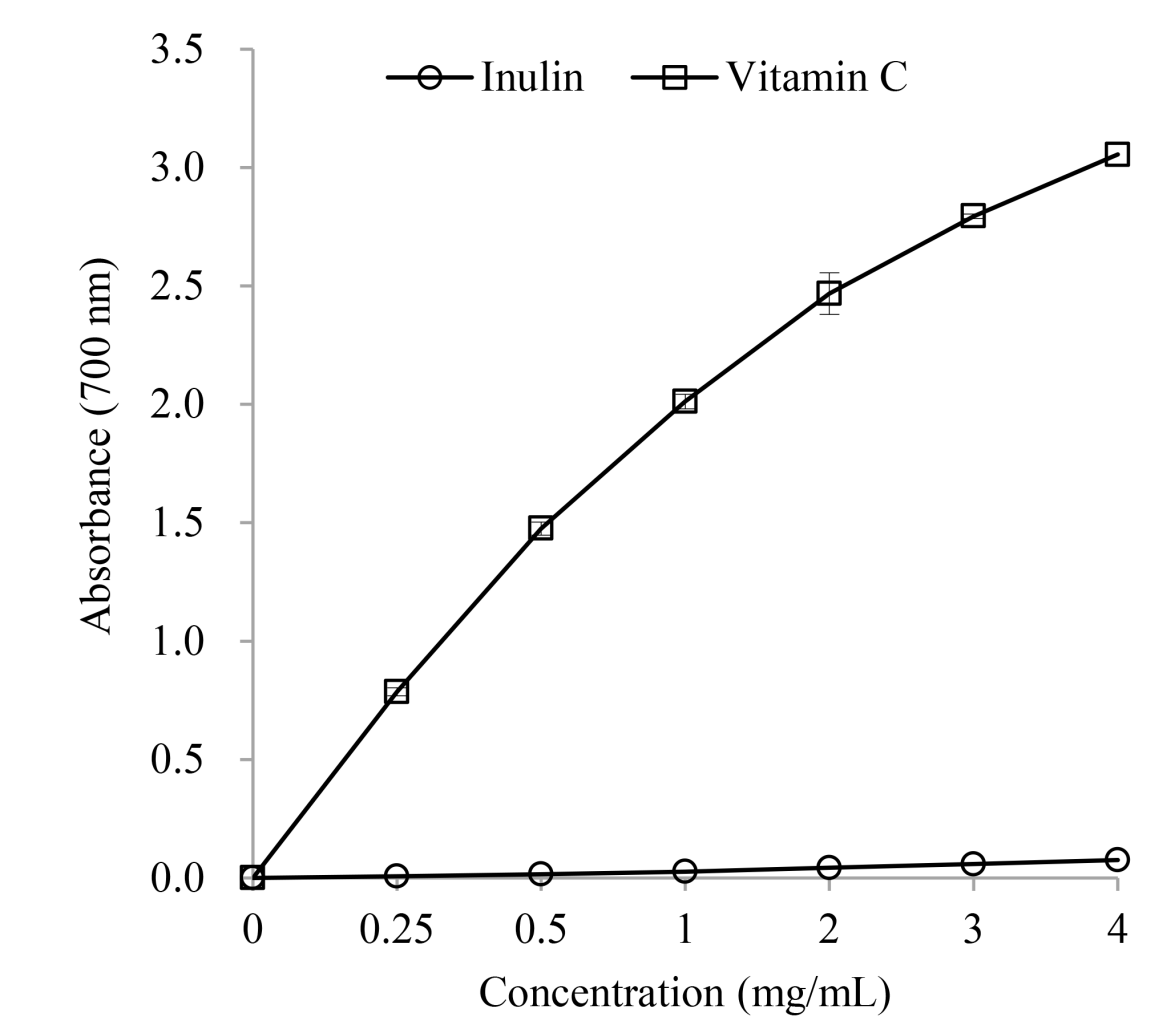
Ferric reducing power of inulin.

The ferric reducing ability of inulin is presented in Fig 3. A dose-dependent reducing power of inulin was observed. Inulin showed a good linear connection (*R*^2^ = 0.989, *P* < 0.05) to concentration in the range from 0.25 to 4.0 mg/mL. However, inulin showed significantly lower ferric reducing power than Vitamin C at doses of detected concentrations (*P* < 0.05).

The results of the antioxidant activity *in vitro* assays indicated that the antioxidant activities of inulin, such as the DPPH radical scavenging activity, ABTS scavenging activity and ferric reducing power were weak and significantly lower than those of Vitamin C (*P* < 0.05). The reason might be due to the molecular characteristics of inulin. The *in vitro* antioxidant activities of polysaccharides were connected to the reductive hydroxyl group terminals, which could accept and eliminate the free radicals [22]. However, the amount of reductive hydroxyl group terminals on inulin is limited. Therefore, the *in vitro* antioxidant activities of inulin are weak.

### Antioxidant activity *in vivo*

#### Laying performance

The effects of dietary inulin on laying performance are summarized in Table 2. Dietary supplementation with inulin significantly improved egg production and feed consumption of laying hens quadratically (*P*<0.01). However, there were no linear or quadratic differences in egg mass, egg weight, or feed conversion ratio among inulin treatments.

**Table 2:** Effects of dietary supplementation with inulin powder on performance of laying hens during the 8-wk period of the feeding experiment.

Item	Dietary inulin concentration (g/kg)					SEM	*P*-value	
	0	5	10	15	20		Linear	Quadratic
**Egg production (%)**	94.6^b^	95.0^ab^	95.5^a^	95.6^a^	95.2^ab^	0.09	0.008	0.001
**Egg weight (g)**	62.8	63.1	63.1	63.1	62.5	0.21	0.670	0.537
**Egg mass (g/hen per d)**	59.4	60.0	60.3	60.3	59.7	0.22	0.579	0.325
**Feed consumption** **(g/hen per d)**	114.3^b^	115.5^ab^	116.2^a^	116.4^a^	115.2^ab^	0.23	0.112	0.005
**Feed conversion ratio**	1.93	1.93	1.92	1.93	1.91	0.02	0.655	0.726

Means within a row with different superscripts differ significantly (*P*<0.05).

Data are means for 6 replicates of 60 laying hens.

Dose response (linear or quadratic) to different levels of inulin supplementation.

Prebiotics are defined as non-digestible food ingredients that beneficially improve host health by combating undesired bacteria and selectively stimulate the growth and/or activity of healthful bacteria in the intestinal tract [23]. Inulin is one of the most commonly used prebiotics in feed for livestock and poultry [24]. Generally, inulin cannot be hydrolyzed by digestive enzymes in the proximal intestinal tract of humans and monogastric animals because of the glucosidic bond of -(2→1)-linkages [25]. Instead, they are fermented by beneficial bifidobacteria and other lactic acid producing bacteria in the large intestine or colon, thereby enhancing their relative populations [26]. It was reported that gut microflora have a significant influence on host health, nutrition, and growth performance by interacting with the nutrient utilization and gut system development of the host [27]. Reduced numbers of coliforms and increased numbers of *Bifidobacteria* spp. suggest a more favorable intestinal microflora that may improve gastrointestinal function, feed digestibility and animal performance [28]. Inulin use as a dietary supplement in animal feed has been shown to enhance immune parameters, modulate intestinal microbiota, and improve growth performance in poultry and swine [29, 30]. Our previous study showed that the inclusion of inulin in laying hens’ diet conferred increasing *Bifidobacteria* count and decreased coliform bacteria count in the cecum [31]. Therefore, the increased egg production in this study might be due to the prebiotic properties of inulin.

#### Antioxidant status of laying hens

Only measuring the *in vitro* antioxidant ability of inulin was not enough to estimate its antioxidant effects in biological systems. Antioxidant assays *in vivo* could reflect the related biological implications of dietary consumption, such as effects on antioxidant enzymes and oxidation-related metabolic pathways. The effects of dietary supplementation with inulin on antioxidant enzyme activities and MDA content in the serum are presented in Table 3. The activities of SOD, CAT, GSH-Px, and T-AOC in the serum of laying hens increased quadratically (*P*<0.001) with the increased in dietary inulin. Meanwhile, the MDA content in the serum of laying hens was quadratically (*P*<0.001) decreased as the dosage of inulin increased from 0 to 20 g/kg of diet. Among the levels of inulin supplemented in the laying hens’ diet, 15 g/kg of inulin supplementation appeared to produce the highest antioxidant enzyme activities but the lowest MDA content in the serum of laying hens.

**Table 3:** Effects of dietary supplementation of inulin powder on serum antioxidant status of laying hens during the 8-wk period of the feeding experiment.

	Dietary inulin concentration (g/kg)						*P*-value	
Item	0	5	10	15	20	SEM	Linear	Quadratic
SOD (U/mL)	170.7^d^	172.7^d^	192.0^c^	214.4^a^	203.8^b^	3.28	0.001	<0.001
CAT (U/mL)	4.83^d^	5.33^c^	5.74^b^	6.45^a^	6.31^a^	0.12	0.002	<0.001
GSH-Px (U/mL)	1373^d^	1502^c^	1593^b^	1716^a^	1597^b^	22.3	<0.001	<0.001
T-AOC (U/mL)	6.02^d^	6.14^d^	6.46^c^	7.76^a^	7.20^b^	0.13	0.001	<0.001
MDA (nmol/mL)	9.18^a^	8.52^b^	8.32^b^	7.09^d^	7.89^c^	0.13	0.001	<0.001

Means within a row with different superscripts differ significantly (*P*<0.05).

Data are means for 6 replicates of 18 laying hens.

SOD, superoxide dismutase; CAT, catalase; GSH-Px, glutathione peroxidase; T-AOC, total antioxidant capacity; MDA, malondialdehyde.

Dose response (linear or quadratic) to different levels of inulin supplementation.

SOD, CAT and GSH-Px are three important endogenous antioxidant enzymes in the antioxidants of the cellular enzymatic system, and they play an important role in antioxidant protection processes [32]. MDA is one of the end products of lipid peroxidation, and the level of MDA can indicate the degree of lipid peroxidation in the body. Liver lipogenesis is significantly improved by estrogens in order to meet the demand for egg production in laying hens. Therefore, ameliorating lipid metabolism, improving antioxidant status, and preventing lipid peroxidation are important for laying hens [33]. Dietary supplementation with inulin increased the activities of SOD, CAT, and GSH-Px and decreased the MDA concentration in the serum, which indicated that inulin could enhance the antioxidant status of laying hens.

The antioxidant process in biological systems involves many aspects of antioxidants, such as digestibility, bioavailability and metabolism [2]. The total antioxidant capacity is recommended for studying antioxidant activity in organisms. T-AOC level indicates the status of the non-enzymatic reactive oxygen species (ROS) defense system. The results of the present study indicated that dietary supplementation with inulin (10, 15 and 20 g/kg) significantly enhanced (*P*<0.05) T-AOC activity in the serum of laying hens (Table 3). This result indicated inulin also had antioxidant capacity related to the non-enzymatic ROS defense system.

The exact mechanisms of the antioxidant abilities of inulin *in vivo* need further research. However, inulin may scavenge ROS indirectly through the generation of antioxidant enzymes and short-chain fatty acids (SCFAs) [34]. One of the possible reasons for inulin’s antioxidant abilities *in vivo* is the prebiotic activity of inulin modifying microflora in the gastrointestinal tract of laying hens. Inulin reaches the large intestine undigested, and it can be fermented by *Bifidobacterium spp*. and other lactic acid-producing bacteria [35]. Our previous study showed that the addition of inulin in the diet conferred increasing *Bifidobacteria* and *Lactobacilli* counts in the cecum of laying hens [31]. It was reported that lactic acid bacteria have SOD, and *in vitro* research has shown that lactic acid itself has the ability to scavenge free radicals. The fermentation of fructooligosaccharide by *Bifidobacteria* leads to the scavenging of free radicals [36]. Additionally, *Lactobacilli* resident in the gut may help the body to decrease the MDA level by releasing its intracellular antioxidant constituents [37]. Another possible reason for inulin’s antioxidant abilities *in vivo* may be due to SCFAs, the anaerobic fermentation products of inulin in the large intestine [38]. It was reported that increased content of butyrate in colonic cells resulted in decreased colonic myeloperoxidase activity and restored glutathione concentration, and butyrate controls the increase in ROS levels [39].

## Conclusions

The present study investigated the *in vitro* and *in vivo* antioxidant activities of inulin. *In vitro* studies showed that inulin had weaker DPPH radical scavenging activity, ABTS radical scavenging activity and ferric reducing power than Vitamin C. *In vivo* studies of laying hens showed that dietary supplementation with inulin significantly improved the antioxidant status of laying hens. The T-AOC and antioxidant enzyme activities of SOD, CAT, and GSH-Px increased, and the MDA concentration in the serum of laying hens decreased. On the basis of the above findings, inulin might have a favorable effect on maintaining or improving antioxidant systems and could be considered a potential antioxidant for the poultry industry. Further research investigating the antioxidant activity mechanisms and utilization of inulin is warranted.

## Supporting information

S1 Dataset Performance of laying hens. (XLS)

S2 Dataset Antioxidant status of laying hens. (XLS)
